# Fully Automated Skull Stripping from Brain Magnetic Resonance Images Using Mask RCNN-Based Deep Learning Neural Networks

**DOI:** 10.3390/brainsci13091255

**Published:** 2023-08-28

**Authors:** Humera Azam, Humera Tariq, Danish Shehzad, Saad Akbar, Habib Shah, Zamin Ali Khan

**Affiliations:** 1 Department of Computer Science, University of Karachi, Karachi 75270, Pakistan; 2 Department of Radiology, University of Michigan, Ann Arbor, MI 48109, USA; thumera@umich.edu; 3 Department of Computer Science, The Superior University, Lahore 54590, Pakistan; 4 College of Computing and Information Sciences, Karachi Institute of Economics and Technology, Karachi 75190, Pakistan; akbersaad@yahoo.com; 5 Department of Computer Science, College of Computer Science, King Khalid University, Abha 61421, Saudi Arabia; habibshah.uthm@gmail.com; 6 Department of Computer Science, IQRA University, Karachi 71500, Pakistan; dr.m.zamin@iqra.edu.pk

**Keywords:** deep learning, fully automated skull stripping, brain magnetic resonance images, MRI, region-based segmentation

## Abstract

This research comprises experiments with a deep learning framework for fully automating the skull stripping from brain magnetic resonance (MR) images. Conventional techniques for segmentation have progressed to the extent of Convolutional Neural Networks (CNN). We proposed and experimented with a contemporary variant of the deep learning framework based on mask region convolutional neural network (Mask–RCNN) for all anatomical orientations of brain MR images. We trained the system from scratch to build a model for classification, detection, and segmentation. It is validated by images taken from three different datasets: BrainWeb; NAMIC, and a local hospital. We opted for purposive sampling to select 2000 images of T1 modality from data volumes followed by a multi-stage random sampling technique to segregate the dataset into three batches for training (75%), validation (15%), and testing (10%) respectively. We utilized a robust backbone architecture, namely ResNet–101 and Functional Pyramid Network (FPN), to achieve optimal performance with higher accuracy. We subjected the same data to two traditional methods, namely Brain Extraction Tools (BET) and Brain Surface Extraction (BSE), to compare their performance results. Our proposed method had higher mean average precision (mAP) = 93% and content validity index (CVI) = 0.95%, which were better than comparable methods. We contributed by training Mask–RCNN from scratch for generating reusable learning weights known as transfer learning. We contributed to methodological novelty by applying a pragmatic research lens, and used a mixed method triangulation technique to validate results on all anatomical modalities of brain MR images. Our proposed method improved the accuracy and precision of skull stripping by fully automating it and reducing its processing time and operational cost and reliance on technicians. This research study has also provided grounds for extending the work to the scale of explainable artificial intelligence (XAI).

## 1. Introduction

We propose a new skull stripping method for brain image analysis. Skull stripping removes non-brain tissues from MR images, but it is hard because of brain variability, noise, artifacts, and pathologies. The existing methods are slower and limited to a single orientation, mostly axial. Our proposed and experimented method uses the modern and robust architecture of deep learning neural networks, viz., Mask–RCNN to learn, detect, segment, and to apply the mask on brain features and patterns from many thousands of brain MR images. This research study improved brain image analysis to help clinical and scientific uses.

The brain, as part of the central nervous system, consists of cerebrospinal fluid and fats. The skull is the strongest bone that protects the brain [1]. Skull stripping is the process of identifying and removing the non-brain tissue from brain MR images wherein skull stripping separates brain images from non-brain parts [2] for better diagnostics of the brain. It plays a crucial role in the pre-processing steps of brain image analysis. Accurate skull stripping is essential for different applications such as brain segmentation, registration, and localization of brain lesions.

### 1.1. Traditional Techniques

Traditional techniques of skull stripping, include Brain Extraction Tools, Brain Sur-face Extraction, and Robust Brain Extraction (ROBEX) [3,4]. BET is a widely used method for skull stripping based on intensity thresholding and morphological operations [5]. BSE is a semi-automated method that uses a deformable surface model to segment the brain from non-brain tissue [6]. The ROBEX algorithm uses intensity thresholding, mathematical morphology, and a multi-scale watershed transform to extract the brain tissue from non-brain tissue [3]. Otsu’s method is a non-parametric approach for image segmentation and is an attractive alternative to the Bayes decision rule [7].

### 1.2. Artificial Intelligence-Based Techniques

The first quarter of the 21st century has been the era of artificial intelligence (AI), and more is expected in the forthcoming decades. Contemporary techniques are more effective and efficient for skull stripping, especially in large lesions or with significant anatomical abnormalities [8,9]. Such methods can robustly deal with variations in image contrast and noise, but may require enormous amounts of training data and therefore greater computational cost [10]. Machine learning (ML)-based AI models of skull stripping have evolved through Deep Learning Neural Networks (DLNN) such as Convolutional Neural Networks (CNN). AI experts have utilized the cutting-edge technology of DLNN to train models for medical image processing using chest X-ray images for the detection of COVID-19 [10]. CNN has revolutionized the field of image processing based on the principle of feature extraction. The CNN-based models progress through Region-based Neural Networks (RCNN) followed by Faster RCNN to today’s state-of-the-art neural networks, viz., Mask–RCNN [11,12].

### 1.3. Machine Learning

Machine learning has contributed significantly to the processing of brain MR images by segmenting and contouring different brain structures and regions of interest [13]. There are at least four types of ML-based brain MR image processing techniques, viz., supervised, unsupervised, semi-supervised, and deep learning. Supervised ML algorithms establish learning from annotated images, where MRI experts manually delineate the brain regions [14]. Support Vector Machines (SVMs) and Convolutional Neural Networks (CNNs) are popular ML-based supervised techniques. Unsupervised ML algorithms discover hidden patterns within brain MR images by identifying similarities and differences in intensity, texture, or shape, and grouping similar voxels or regions [15]. K-means and Gaussian Mixture Models (GMMs) are popular ML-based unsupervised techniques. Semi-supervised ML algorithms use a small amount of annotated data to guide the segmentation process while taking unlabeled data to learn more generalizable features. Deep learning models such as CNNs can automatically learn hierarchical features from raw image data [16,17]. Popular deep learning architectures include U-Net, Fully Convolutional Networks (FCNs), and U-Net.

### 1.4. Deep Learning

Deep learning has revolutionized the processing, segmentation, and identification of regions in brain MR images [13]. ResNet and U-Net are the most popular models of deep learning. ResNet is known for its residual connections and achieves high accuracy in tasks such as brain tumor segmentation [18]. U-Net precise localization has been extensively applied to segment brain MR images [19]. Many researchers have combined ResNet and U-Net to leverage their respective strengths for enhanced accuracy and precision [20]. In contrast, the challenges related to such architectures include class imbalance and limited training data [21]. Their application has advanced brain MR image analysis including anatomical structure identification [22].

### 1.5. CNNs and RCNNs

CNN consists of convolutional layers that apply filters to input images and generate feature maps [23]. Non-linear activation functions and pooling layers follow the CNN for down sampling. RCNN combines CNN with recurrent layers such as LSTM or GRU to capture temporal dependencies in sequential data [24]. RCNN effectively models both spatial and temporal information, making them valuable for tasks involving sequential or spatiotemporal understanding.

### 1.6. Mask–RCNN

Region-based Neural Network (RCNN) uses selective searching to identify regions of interest (ROIs) and applies a convolutional neural network to classify ROIs as foreground or background [25]. Faster RCNN, an extended version of RCNN, uses a Region Proposal Network (RPN) to generate ROIs by adding more CNNs [26,27].

Nonetheless, Mask–RCNN is the most modern type of DLNN, an extended version of Faster RCNN by adding a mask branch, which generates binary masks for each ROI. Mask–RCNN masks the detected object [24,28,29]. Mask–RCNN applies Residual Neural Network (ResNet) [30] architecture for feature extraction from the input image, which comprises cerebral cortex pyramidal cells and uses jumps over applied layers [31]. ResNet works as the backbone architecture of the Mask–RCNN [32]. It also applies Region Proposal Network (RPN) to predict the presence or absence of the targeted object [24,33]. It forwards feature maps having target objects. Pooling layers make uniform shapes of received featured maps to connect layers for further processing [34,35]. Eventually, the fully connected convolutional layers receive feature maps to predict class labels and coordinates of bounding boxes [28,31]. Mask–RCNN computes Regions of Interest (RoIs) with a benchmark value = 0.5 [36] for declaring the regions as RoIs, followed by the addition of the mask branch to apply a mask on established RoI [37].

The application of Mask–RCNN contours object detection, segmentation, image captioning, and instance segmentation [24,38]. It has achieved state-of-the-art performance in various benchmark datasets [39]. In object detection, Mask–RCNN has shown excellent performance in detecting multiple objects with high accuracy. Instance segmentation uses individual instances of objects in an image and provides a more detailed understanding of the scene. In image captioning, Mask–RCNN generates captions to describe target objects and their locations.

**Figure 1:**
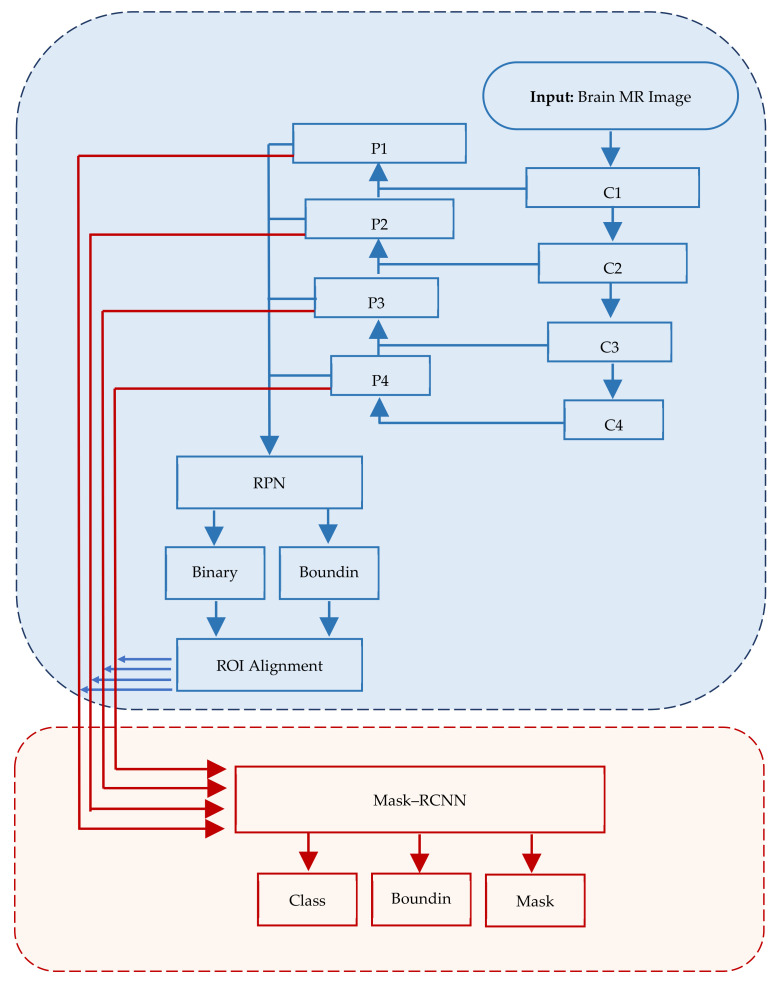
Mask–RCNN architecture comprising three major segments, viz., input, regions of interest and application of mask.

Mask–RCNN offers more precise and accurate results [40]; however, in some cases, it takes around 48 h to train the system. Numerous public databases such as Common Objects in Context (COCO) are available with training weights to train systems using the transfer learning approach [41,42]. Overall, Mask–RCNN is an effective tool for image analyses and has the potential for further advancements in computer vision. Figure 1 depicts the structure of Mask–RCNN.

In Mask–RCNN architecture, the input image passes through convolutional layers (C1, C2, …, Cn) while extracting feature maps (P1, P2, …, Pn) at each layer. RPN is applied on each ex-traced feature map. 

## 2. Research Gap

Mask–RCNN is the latest DLNN-based method for image segmentation. Literature suggests that learning weights of numerous objects and classes for the training of DLNN are readily available [28,43]. Extensive literature did not provide sufficient empirical evidence about utilizing Mask–RCNN for skull stripping from brain MR images [43]. Moreover, renowned public libraries such as COCO do not have training weights to train systems for predicting the brain and skull stripping from the given brain MR image [44]. This research study has bridged such a research gap by stripping the skull from brain MR images and exploring the right mix of learning weights to utilize for future training. The application of Mask–RCNN may leverage the power of deep learning by limiting the demerits of conventional skull stripping methods. The ability of Mask–RCNN to precisely delineate the boundary of the skull and separate it from brain tissues could greatly enhance the accuracy and reliability of subsequent neuroimaging analyses.

## 3. Methodology and Experiment on Mask–RCNN for Skull Stripping

We opted for experimental design and the philosophic lens of pragmatism, which freed us from choosing a workable set of methods that may provide the desired results rather than rigid methodological options [45,46]. Pragmatism focuses on the practical outcomes and gives freedom to apply methods and systems that offer the best results, rather than too much reliance on metaphysical and ontological beliefs [47]. We employed the trial and error approach wherein we tested, retested, and fine-tuned different hyperparameters to obtain the desired outcome.

We retrieved data from three libraries, viz., BrainWeb, NAMIC, and a local hospital for training and testing the system. We created a ground truth and conducted baseline experiments with two state-of-the-art traditional methods, Brain Extraction Tool (BET) and Brain Surface Extraction (BSE). We augmented data by cropping, resizing, rotating, flipping, and noising the input images to increase the size of input data as well as to train the system on highly diversified images. We created a ground truth mask and retrieved annotated values using the VGG Image Annotator (VIA) for training purposes. We provided input to the system for training of Mask–RCNN using ResNet–101 architecture. The system extracted features from the input brain MR images. In the next stage, the system created anchor boxes using Region Proposal Networks (RPNs). The system created bounding boxes by regressing Regions of Interest (ROIs); based on this, the system detected the brain as an object using Non-Max Suppression (NMS). In the next level, the system segmented the detected brain and overlaid a mask on the detected region. 

**Figure 2:**
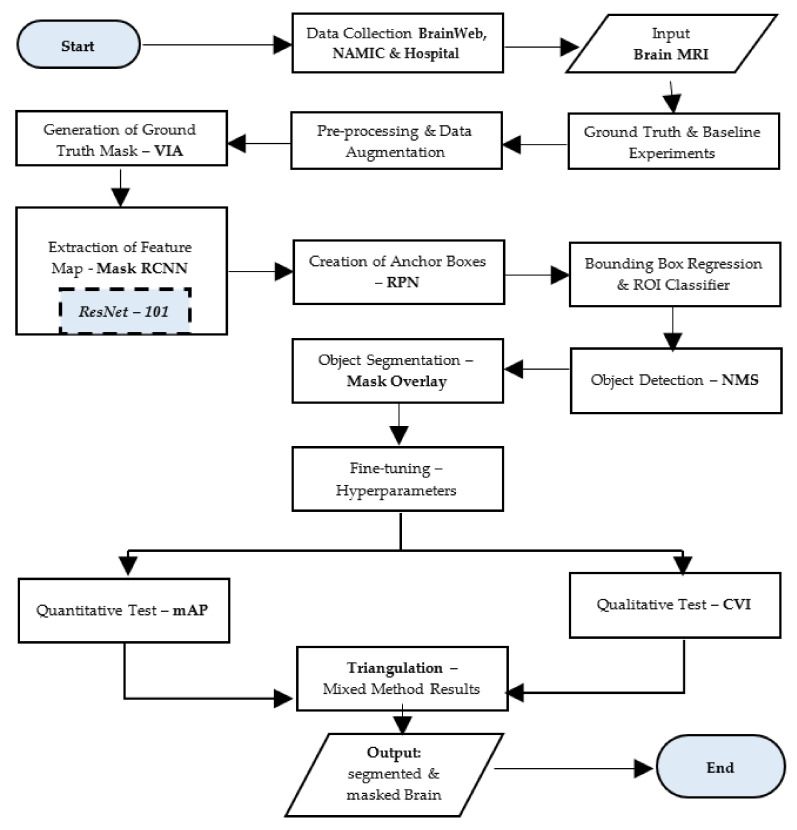
Stages of experiment.

In the next phase, we fine-tuned the hyperparameters to obtain the best possible results from the Mask–RCNN. Once the best possible results are generated, we compared the quantitative output using Mean Average Precision (mAP) with the qualitative output using content validity index (CVI). The mAP is a widely accepted and used metric to gauge the accuracy of object detection and image segmentation [48,49]. The CVI is a robust technique to measure content validity through empirical observation by the field expert [50]. This comparison is called a mixed-method triangulation of results. The result of the entire process is in the form of a segmented mask that represents the brain area without a skull. Figure 2 depicts ten rigorous stages of the experiment:

### 3.1. Data Collection

**Figure 3:**
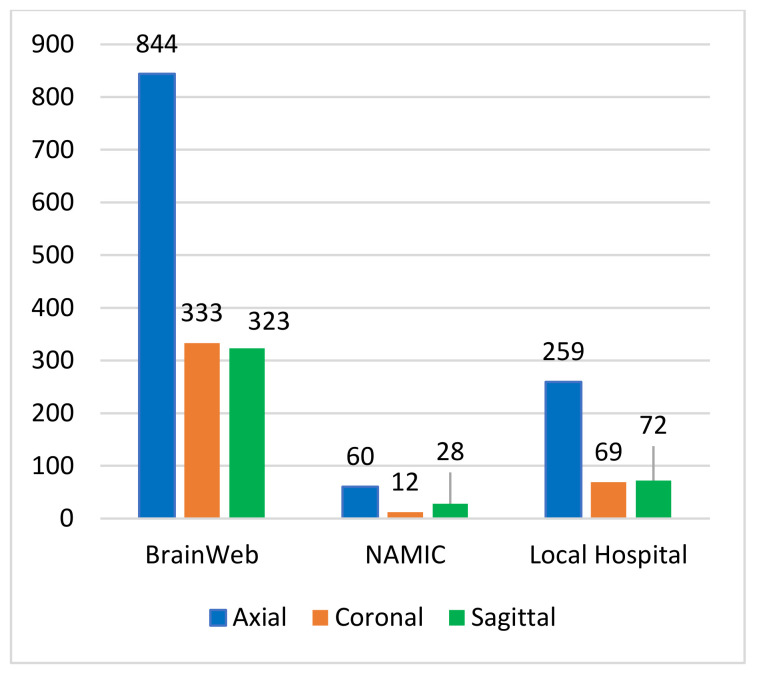
Anatomical orientation of brain MR images retrieved from different sources.

We compiled a dataset with 2000 healthy brain MR images retrieved from two public libraries, BrainWeb [51] and NAMIC [52], and one internationally renowned hospital. The modality type of all selected brain MR images is T1. Figure 3 depicts the contribution of brain MR images from different databases:

We retrieved a total of 1500 brain MR images from BrainWeb, of which 844 were axial, 333 were coronal, and 323 were sagittal. In contrast, we retrieved 100 brain MR images from NAMIC, of which 60 were axial, 12 were coronal, and 28 were sagittal. However, we retrieved 400 brain MR images from a well-reputed local hospital, of which 259 were axial, 69 were coronal, and 72 were sagittal.

### 3.2. Ground Truth and Baseline Experiments

**Figure 4:**
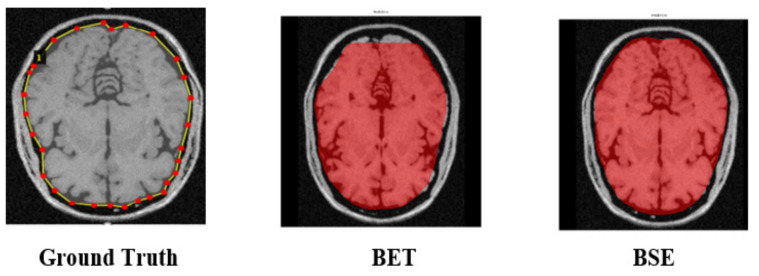
Generation of baseline segmentation results for Ground Truth and comparison of results.

We employed two state-of-the-art image segmentation methods, viz., BET and BSE for generating baseline values of brain MR images to understand the effectiveness and precision of our experimented method. BET is a traditional method widely used for skull stripping. It segments the brain tissues from the non-brain tissues by using features such as intensity, shape, and contrast of brain MR images [53]. BSE is also a traditional method that uses several filters, edges, detection, and morphological operations to extract the surface of brain MR images [6]. We created ground truth (GT) by contouring the cerebrum area using polygonal labeling. Moreover, we segmented the same brain MR image using BET and BSE techniques. We treated selected brain MR images to remove noise because both baseline methods require noise-free images. BET utilizes a deformable model to segment the input MR image while using a non-linear smoothness technique for noise treatment. BSE combines edge detection and morphology-based techniques to segment the input MR image using an anisotropic diffusion technique for noise treatment. Contrary to this, the Mask–RCNN-based experimental method does not require noise treatment. Figure 4 depicts the output of baseline segmentation.

### 3.3. Pre-Processing and Data Augmentation

**Figure 5:**
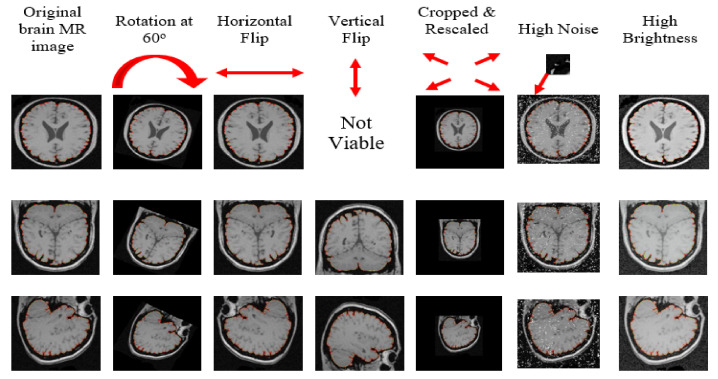
Augmentation of brain MR images reserved for training.

We divided brain MR images into three groups each for one stage of the experiment (training (75% = 1500 images), validation (15% = 300), and testing (10% = 200)) using the multistage random sampling technique elaborated previously, which ensured a fair proportion of all source-databases and anatomical orientations of the already selected 2000 images. Moreover, we augmented brain MR images by rotating, flipping, scaling, noising, and brightening, which offered a larger dataset with higher diversity to ensure the generalizability of Mask–RCNN. Such treatment also provided ground truth for the training of Mask RCNN to increase its capability for detecting objects with different orientations. The data augmentation of 1500 images provided 8122 augmented brain MR images as the system transformed each of the 878 axial images into 5 augmented images. Moreover, it transformed each of the 303 coronal images and 319 sagittal images into 6 augmented images. Figure 5 depicts the output of augmentation of brain MR images.

### 3.4. Generation of Ground Truth Mask

**Figure 6:**
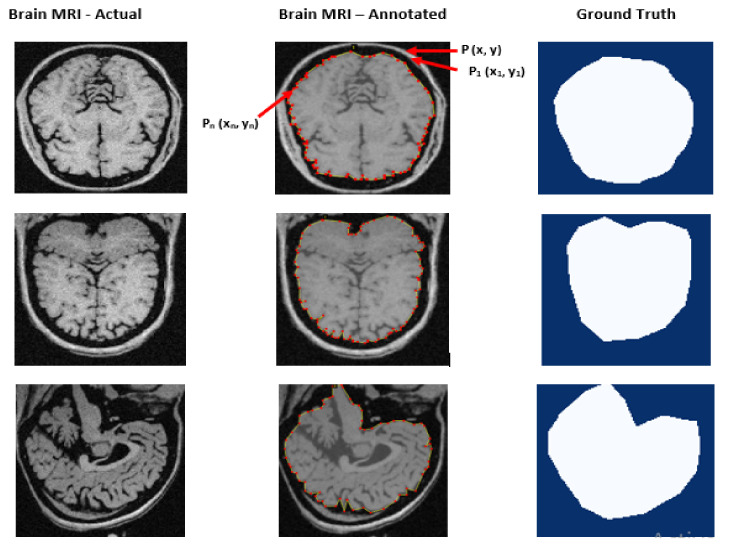
Ground truth mask created with the golden standard.

We utilized the VGG Image Annotator (VIA) to create golden standard ground truth masks by applying a geometrical polygonal method for annotating the image and labeling objects. As a result, we created a polygonal shape by connecting points P1, P2, P3 … Pn, which contoured the entire brain cerebrum. The contouring polygon has the x and y coordinates stored in an annotation file. We created said file for uploading in the system so it could identify polygonal contours to detect the image within it as a ground truth. Eventually, the system picked all x and y coordinates of all input images for training purposes. Figure 6 depicts the output of the ground truth mask created using the golden standard.

### 3.5. Extraction of Feature Maps

**Figure 7:**
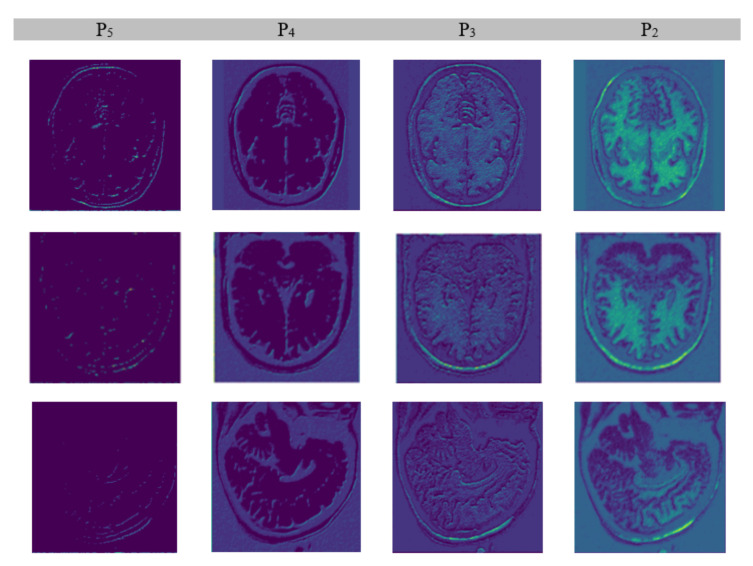
Extraction of features.

We extracted features from the input brain MR images by applying a convolutional neural network equipped with ResNet-101-FPN architecture. ResNet–101 comprises 101 convolutional layers wherein initial layers extract edges while terminal side layers extract brain regions. Moreover, terminal side layers also convert images into feature maps. The process works in a structure of three dimensions from the bottom to top, from the top down, and to horizons making it robust. The bottom-to-top dimension has convolutional layers from C1 to C5; the top-to-bottom dimension has layers from P2 to P5, while a horizontal connection of 3 × 3 kernel having a double effect (2×) of P* and C* exists in between both vertical dimensions. The refining of feature maps worked from P5 to P2. The inner-most layer (P2) created the best feature map. Figure 7 depicts the output of the feature extraction process.

### 3.6. Creation of Anchor Boxes

**Figure 8:**
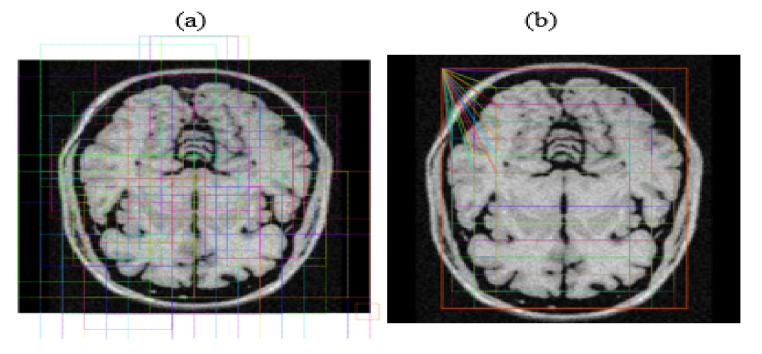
(**a**) Positive anchor boxes; (**b**) refined and regressed anchor boxes.

**Figure 9:**
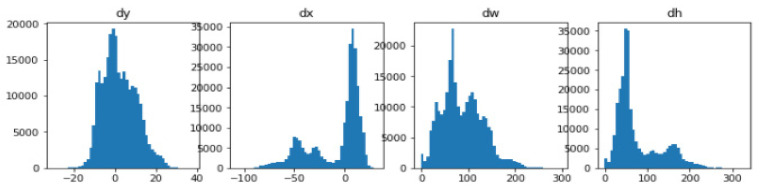
Histograms of derivatives taken from Region Proposal Network (RPN) bounding boxes deltas.

The system identified Regions of Interest (RoIs) having targeted objects with significantly high probability from extracted feature maps by implementing a Region Proposal Network (RPN). RPN scanned brain MR images using the sliding-window technique to detect targeted objects. Rectangular boxes, called anchor boxes, cover a large part of the targeted object. Several weights applied on anchor boxes produced two outputs, classes and boxes. In this study, the anchor class is binary, either positive or negative. The positive response represents the presence of the brain inside anchor boxes, while the negative response represents background information such as non-brain parts. We derived the anchor class by calculating the intersection over the union (IoU) of ground truth with the anchor box. The threshold value for acceptable IoU ≥ 0.7, which shows that all positive anchors meet the benchmark criteria. Consequently, object classification transferred only positive anchor boxes with the brain in the foreground. At this level, the probability coefficient of each anchor box shows a high chance for occurrence of the brain. RPN further refined anchor boxes, shifting their position slightly and resizing them a little to fit over the brain. RPN takes derivative values to estimate changes in the anchor box. Figure 8 depicts two outputs wherein (a) shows positive anchor boxes and (b) shows refined and regressed anchor boxes, while Figure 9 depicts a histogram of derivatives of Region Proposal Networks.

We observed that several positive anchor boxes are overlapping. We applied non-max suppression (NMS) to retain anchors boxes to reduce redundancy with a high probability ≥ 0.85. This reduced processing time and increased the prediction accuracy.

### 3.7. Bounding Box Regression and ROI Classifier

**Figure 10:**
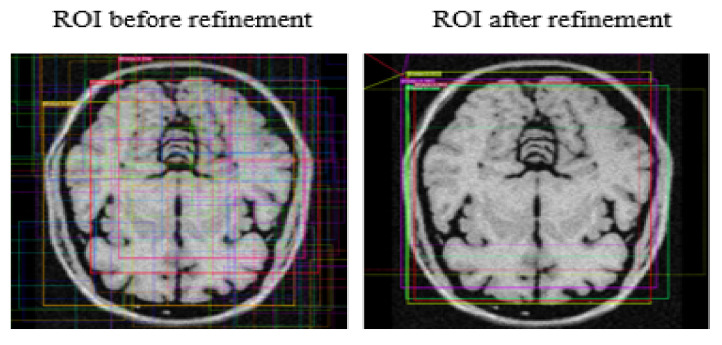
ROI—Classification before and after refinement.

We applied classifier heads to classify regions already proposed by RPN. This process generated probabilities for each class and regressed bounding boxes to refine them further. Eventually, the system created a mask head according to the regression scores of bounding boxes. In this research study, brain MR images contain binary classes; due to this, the system classified the brain as a foreground object and the non-brain part as a background object. The ROI classifier logically retains the foreground and discards/ignores the background object. Regression applied on high-probability anchor boxes refined their spatial position to encapsulate already classified foreground objects (brain). The ROI classifier does not effectively handle input varying in size; therefore, a function of ROIPooling crops input to adjust its functionality, followed by ROIAlign that takes samples of different feature maps at multiple points using the bilinear interpolation technique. Figure 10 depicts the output of the ROI—classification before and after refinement.

### 3.8. Object Detection by Applying NMS

**Figure 11:**
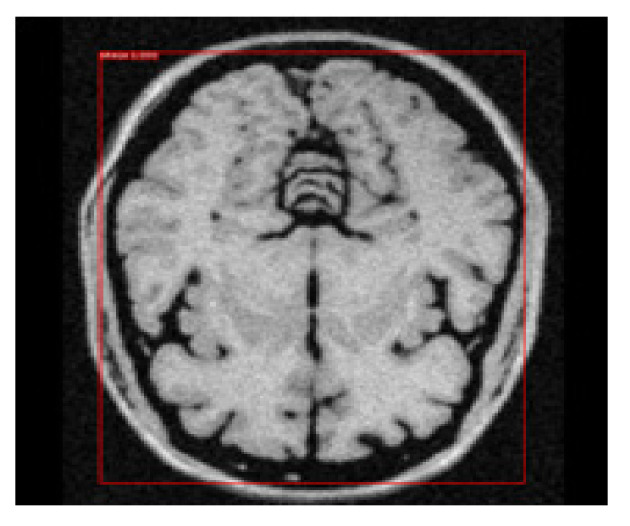
Retention of final bounding box with object detection.

We re-applied the function of NMS to deduce the bounding box with the highest probability from the remaining positive anchor boxes. We set the suppression threshold value using IoU ≥ 0.85 and opted for the maxima approach to retain the positive anchor box with the highest probability. This step completes the process of the first objective of this study to detect the brain encapsulated in the final remaining bounding box. Figure 11 depicts the output of the bounding box retained for object detection.

### 3.9. Object Segmentation by Generating Overlay Mask

**Figure 12:**
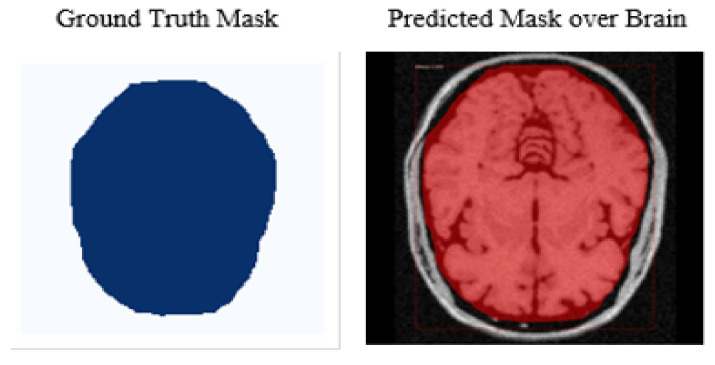
Mask prediction on the brain region.

**Figure 13:**
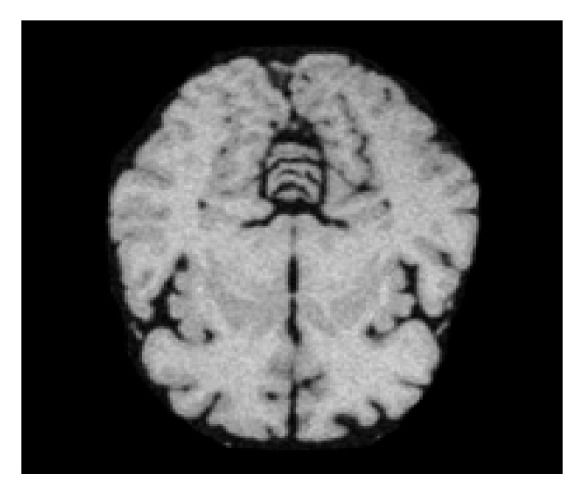
Skull-stripped brain MR Image.

The ultimate objective of this research study is to apply a mask over the detected brain for segmentation purposes. We masked the segmented part using the sigmoid function on each pixel of the detected brain. The convolutional network generates masks from positive regions already selected by the ROI classifier. We had already created ground truth in earlier stages of experiments; therefore, the system predicted applying a mask using a low resolution of 28 × 28 to ensure the minimum memory usage. The low pixel resolution of the masked image shows its lighter layer. Therefore, we scaled down the ground truth ROI to 28 × 28 while padding it with 0 to avoid distortion or disruption. After predicting overlay masks, we scaled their resolution up for inference purposes. The targeted region, the skull-stripped brain, exists under an overlay mask. Finally, we have concluded that the skull stripping from brain MR images using a deep learning technique of Mask–RCNN. Figure 12 depicts the output of the ground truth mask and predicted mask over the brain, while Figure 13 shows a skull-stripped brain MR image.

### 3.10. Fine Tuning of Hyper-Parameters

We fine-tuned the trained model through pre-assigned hyperparameters to enhance the preciseness of skull stripping. We devised three training models with a predefined combination of relevant hyperparameters, except for the learning rate and the number of epochs. We used the trial and error method to find the best model. Table 1 presents hyperparameters with the same values for all three training models, while Table 2 presents hyperparameters that are different among models.

**Figure 14:**
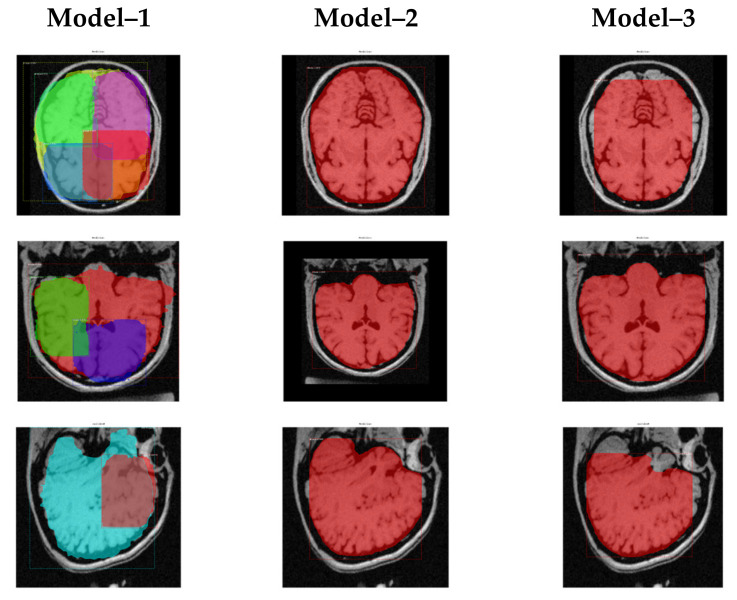
Generation of overlay mask.

**Figure 15:**
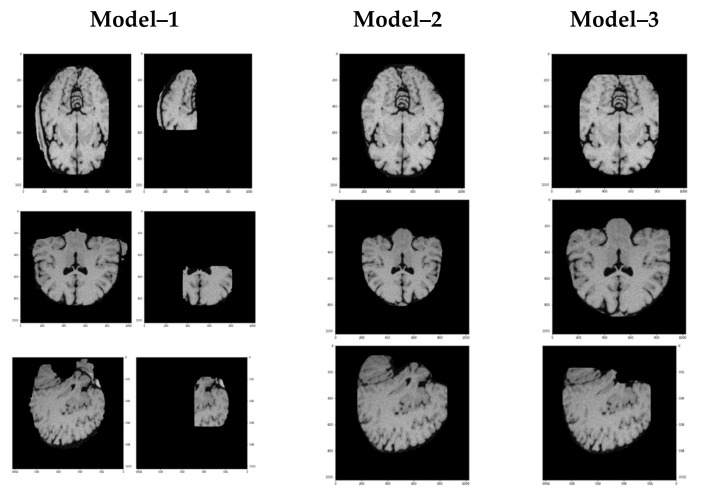
Brain extraction by all three trialed models.

We trained all three training models from scratch by providing a ground truth and generated training weights. Model 1 is the fastest in training because it has only 100 epochs with the highest pre-set learning rate. Model 2 is slower than Model 1 because it has five times more epochs and half the learning rate of Model 1. Model 3 is the slowest among all three models because it has ten times more epochs while the learning rate is 10% of Model 1. Figure 14 presents the results of overlay masks, whereas Figure 15 depicts skull-stripped brains extracted from MR images.

Model 1 underperformed and applied many masks, while Model 3 also underperformed but applied one mask per image. Model 2 performed up to the mark with the best results, and the masked area fits correctly on the brain. Therefore, we adopted the hyperparameter values of Model 2 as the best fit for skull stripping from brain MR images.

### 3.11. Precision–Recall Curve

We also measured the performance of the proposed Mask–RCNN using a precision–recall curve. It is a robust technique to measure the success of prediction by the trained system especially when the structure of objects is complex. The precision represents the area of true positives out of the total positive including true positive and false positive while the recall represents an area of true positives out of the total of true positives and false negatives. The following is the standard formula to calculate precision and recall values based on true positive (TP), false positive (FP), and false negative (FN):

$$Precision=\frac{TP}{TP+FP}\;\;\;\;\;\;\;\;Recall=\frac{TP}{TP+FN}$$

**Figure 16:**
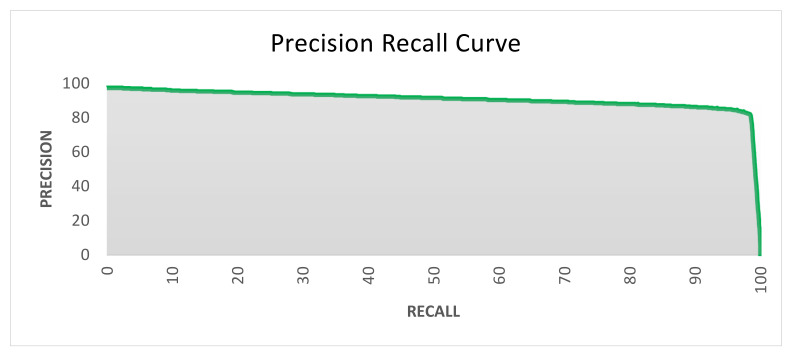
Precision–recall curve of prediction by the trained Mask–RCNN.

The following Figure 16 presents the precision–recall curve, which is quite acceptable with good consistency with higher precision and lower recall. 

## 4. Results and Discussion

We calculated Mean Average Precision (mAP) to measure the accuracy and precision of our trained and experimented Mask–RCNN. The coefficient of mAP is a contemporary and valid measure to quantify precision and accuracy from segmented images. The mAP coefficients, AP0.05 = 0.91 and AP0.95 = 0.97 are outstanding for a fully automated deep learning-based skull stripping method. Table 3 presents a comparative summary of descriptive statistics of the mAP coefficient scored by Mask–RCNN with two state-of-the-art traditional methods, BSE and BET.

The average mAP of 200 brain MR images uploaded for testing the trained model is 0.93, which is much better. The range (maxima–minima) is merely 0.07, average mAP = 0.93, and standard deviation = 0.022, significantly better than the corresponding coefficients of BSE and BET. The weakest prediction accuracy by Mask–RCNN is 0.89, compared to BSE = 0.86 and BET = 0.85. Therefore, Mask–RCNN has outperformed BSE and BET on the scale of mAP. Table 4 presents the result of the mAP coefficient of 10 randomly selected brain MR images and processes with Mask–RCNN, BSE, and BET.

The presented table shows that Mask–RCNN has outperformed traditional competitive methods in the case of each brain MR image. Except for the mAP coefficient, Mask–RCNN has an advantage over competitive methods as it is fully automated and does not need human assistance for execution. Once it has generated training loads, we can transfer them to other experiments on the same object (brain), which is called transfer learning.

## 5. Conclusions

The objective of this research study was to conceptualize, train, and test a deep learning-based approach to fully automate the skull stripping mechanism for better diagnosis and that may also help reduce the time of brain-related medical issues with increased accuracy and precision. Therefore, we reviewed the literature and learned that Mask–RCNN is the most robust technique available but lacks evidence about its application on brain MR images for skull stripping. Therefore, we opted for Mask–RCNN, trained it from scratch, generated learning weights, and tested it rigorously on brain MR images of different thicknesses, noise, intensity, and orientation. We used mAP as a tool to measure the accuracy and precision of output generated by Mask–RCNN. Eventually, the performance of Mask–RCNN outperformed BSE and BET.

### 5.1. Research Significance and Practical Implications

The research study is fruitful for business organizations manufacturing medical diagnostic equipment. They can develop Mask–RCNN-based machines to decrease time and operating costs, and increase the accuracy and precision of diagnostics by medical practitioners. The diagnostic experts may use it as a silver-standard method, parallel to the golden standard for corroboration of results generated by both, which shall surely decrease the probability of error in diagnosing medical issues.

### 5.2. Compliance with Ethical Standards

We used data imported from public libraries and codified and executed the entire experiment in legal software. We did not use plagiarized content in the research study. However, we acknowledged understanding learned from other studies by citing them appropriately.

## Figures and Tables

**Table 1:** Hyper-parameters with the same Values for all three models.

Configuration	Pre-Set Value
Backbone Layer	ResNet
Steps per Epoch	100
The Momentum of the Learning Rate	0.9
Minimum Dimension of Image	256

**Table 2:** Hyperparameters are different for fine-tuning.

Configuration	Model		
	1	2	3
Number of Epochs	100	500	1000
Learning Rate	0.001	0.0005	0.0001

**Table 3:** Descriptive statistical summary of mAP coefficient.

Statistics	Mask–RCNN	BSE	BET
Minima	0.89	0.86	0.85
First Quartile	0.91	0.90	0.89
Median	0.93	0.91	0.91
Third Quartile	0.95	0.94	0.93
Maxima	0.96	0.95	0.94
Mean (Average)	0.93	0.91	0.91
Standard Deviation	0.022	0.047	0.035

**Table 4:** Result of randomly selected brain MR images.

Description of Brain MR Image	Mask–RCNN	BSE	BET
T = 1, N = 0, I = 40, O = AX	0.96	0.91	0.94
T = 3, N = 0, I = 40, O = AX	0.94	0.9	0.90
T = 3, N = 0, I = 20, O = AX	0.90	0.89	0.89
T = 1, N = 0, I = 20, O = AX	0.94	0.88	0.92
T = 1, N = 0, I = 20, O = AX	0.93	0.90	0.91
T = 1, N = 0, I = 40, O = CR	0.95	0.91	0.93
T = 3, N = 3, I = 20, O = CR	0.93	0.88	0.90
T = 1, N = 0, I = 20, O = CR	0.94	0.92	0.91
T = 1, N = 1, I = 20, O = SG	0.91	0.87	0.89
T = 1, N = 0, I = 20, O = SG	0.92	0.91	0.90

T = Thickness, N = Noise, I = Intensity, O = Orientation, AX = Axial, CR = Coronal, SG = Sagittal.

## Data Availability

No new data was created for this research study.
